# Central concepts in mental health promotion programs for children and adolescents: Evidence and examples from Switzerland

**DOI:** 10.1093/eurpub/ckac129.451

**Published:** 2022-10-25

**Authors:** F Wieber, A Zysset, A von Wyl

**Affiliations:** 1 Institute of Public Health, ZHAW Zürich University of Applied Sciences, Winterthur, Switzerland; 2 Department of Psychology, University of Konstanz, Konstanz, Germany; 3 Clinical Psychology and Health Psychology Section, ZHAW Zürich University of Applied Sciences, Winterthur, Switzerland

## Abstract

Universal mental health promotion programs draw on a wide range of concepts to promote children's and adolescents’ mental health. This presentation aims to provide an overview of the central concepts from health, developmental and social psychology, psychiatry, and public health/health promotion and to illustrate their application using examples from Swiss mental health promotion programs for children and adolescents. Concepts on internal, biological, psychological and psychosocial factors such as the ten life skills that are recommended by the WHO, self-efficacy, (mental) health literacy, and the resilience literature are looked at as well as concepts on external risk and protective factors such as social support (quality of attachment and relationships, positive family climate and peer relations) and the quality of educational institutions. Evidence on their effectiveness is reported and conceptual similarities and differences as well as the underlying mechanisms of action are explored from a health behavior change perspective using the behavior change wheel. Finally, implications for the design and implementation of mental health promotion programs are discussed

